# Curcumin protects radiation-induced liver damage in rats through the NF-κB signaling pathway

**DOI:** 10.1186/s12906-020-03182-1

**Published:** 2021-01-06

**Authors:** Wei Li, Liangjun Jiang, Xianzhou Lu, Xianrong Liu, Mingjiong Ling

**Affiliations:** 1 Department of Hepatobiliary Surgery, Affiliated Nanhua Hospital, University of South China, Hengyang, 421002 Hunan China; 2 Department of Gastroenterology, Affiliated Nanhua Hospital, University of South China, Hengyang, 421002 Hunan China

**Keywords:** Curcumin, Radiation-induced liver damage, Oxidative stress, NF-κB pathway, Apoptosis, Inflammation

## Abstract

**Background:**

Curcumin has been demonstrated to exert anti-oxidant, anti-fibrotic, anti-inflammatory, and anti-cancer activities. This study was conducted to observe the effect and inner mechanism of curcumin in rats with radiation-induced liver damage (RILD).

**Methods:**

Thirty SD rats were classified into Control, Radiation group and Curcumin (Cur) + Radiation group (*n* = 10 in each group). The changes in body weight of the rats were observed on the 3rd, 7th and 14th days after the treatment with curcumin. On the 14th day post treatment, the heart blood of the rats was drawn for measurement of liver function indices including total protein (TP), alanine aminotransfetase (ALT), alkaline phosphatase (ALP), lactate dehydrogenase (LDH) as well as aspartate aminotransfetase (AST). Subsequently, the rats were euthanized and liver tissues were taken to observe liver morphological changes using hematoxylin-eosin (HE), and to analyze apoptosis condition using transferase-mediated deoxyuridine triphosphate-biotin nick end labeling (TUNEL) assays. Meanwhile, the oxidative stress level in liver tissue homogenate was determined by biochemical analysis. The expression of nuclear factor kappa B (NF-κB) pathway-associated and apoptosis-associated proteins was detected using Western blot analysis, and the expression levels of inflammatory factors were measured by Enzyme-linked immunosorbent assay (ELISA).

**Results:**

The reduced body weight was observed in rats of the Radiation group compared to the Control and Cur + Radiation groups on day 14. In the Radiation group, hepatic cell edema and inflammatory cell infiltration could be visible under the light microscope, and the hepatocytes presented with vacuolar degeneration. In the Cur + Radiation group, the hepatocytes swelled under the microscope, but the pathological changes were alleviated in comparison with the Radiation group. RILD rats with curcumin treatment presented with decreased ALT, AST, ALP, LDH, and maleicdialdehyde (MDA) levels, and elevated TP, superoxide dismutase (SOD), caspase activated DNase (CAD) and glutathione (GSH) levels. Apoptosis and inflammation in rats with RILD were up-regulated, and the NF-κB pathway was activated, but they were reversed after continuously intragastric administration of curcumin for 14 days.

**Conclusion:**

Our study highlights that curcumin treatment reduces the liver damage caused by radiation through the inhibition of the NF-κB pathway.

**Supplementary Information:**

The online version contains supplementary material available at 10.1186/s12906-020-03182-1.

## Background

In both human and animals, radiotherapy plays a role in controlling tumors, while its injuries to the liver, bone marrow and other tissues restrict the radiation dose to suppress tumors [1]. The effects of radiation on the liver are influenced by lifestyle factors, especially diet, alcohol and obesity [2]. Irradiation to the non-tumor part of the liver leads to cell damage, and is termed as radiation-induced liver damage (RILD) [3]. Radiation-induced liver damage (RILD) is histologically featured with venous obstruction, distortion of the lobular architecture, a loss of parenchymal hepatocytes sinusoidal congestions, as well as some clinical symptoms such as ascites, fatigue, and elevated liver enzymes [4]. Currently, no pharmacological therapy is obtainable to relieve RILD. So, it is essential to o identify the toxicity at an early stage using biomarkers or to develop approaches to minimize the toxicity. Natural products intake that is abundant in anti-oxidant phytochemicals is becoming a hot issue, and the products can be considered to be radioprotective in preventing ionizing radiation injury [5].

Curcumin is a phenolic compound extracted from the rhizomes of turmeric (*Curcuma longa*) [6], which is widely applied in clinical therapy due to its safety, tolerability, and cost-effectiveness [7]. A review has suggested that curcumin has therapeutic capabilities in various chronic diseases including diabetes, obesity, cancers, as well as cardiovascular, pulmonary, neurological and autoimmune diseases [8]. In recent years, curcumin has been revealed to protect against liver damage based on the clinical trials of human [9]. Also, curcumin is shown to alleviate the liver damage in some animal models of liver injury, and can be successfully used to treat with liver damage in human and animal models [10–15]. For instance, a prior study has reported that curcumin decreases the aspartate aminotransferase (AST) and alanine aminotransferase (ALT) levels in serum of a model of liver damage in rats [13]. Oral administration of curcumin to BPA-exposed rats reverses the lipid peroxidation product contents, and enhances glutathione peroxidase (GPx) and glutathione S-transferase (GST), catalase (CAT), and superoxide dismutase (SOD) activities to against BPA-stimulated hepatotoxicity in rats [15].

Curcumin can exert its biological functions through a diverse range of molecular targets and signaling pathways such as β-catenin, p38 mitogen-activated protein kinase (MAPK), signal transducer and activator of transcription 3 (STAT3), nuclear factor-κB (NF-κB), nuclear factor E2-related factor 2 (Nrf2), reactive oxygen species (ROS), cyclin D1, cyclooxygenase-2 (COX-2), vascular endothelial growth factor (VEGF), glutathione, and tumor necrosis factor-α (TNF-α) [9]. It is reported that curcumin enables to retard the inflammatory mediators’ production partly via the inhibition of the Wnt/β-catenin and NF-κB pathways as well as nucleotide binding oligomerization domain-like receptor 3 (NLRP3) inflammasome activation [16]. NF-κB is essential in radioresistance together with induced radiosensitivity [17]. Curcumin shows its antioxidant and inhibitory effects against a diverse range of hepatic diseases through the NF-κB signaling pathway [18–20]. Nanji et al. [20] supported that curcumin enables to prevent alcohol-induced liver disease in rats through modulation of the NF-κB signaling pathway. A review detailed the relationship between curcumin and radiotherapy, and addressed the radioprotection of curcumin [21]. Although many studies have focused on RILD for decades, it remains unclear for its inner mechanisms and effective treatments. Therefore, the current study aimed to inquiry into the capabilities and its mechanism of curcumin in rats with RILD.

## Methods

### Ethics statement

This study got approval from the Institutional Animal Ethics Committee of Nanhua Hospital, China, and all animal care and experimental procedures strictly adhered to the *Guidelines for the Care and Use of Experimental Animals* and *Guidelines for Animal Rescue* [22, 23].

### Animals and treatments

Thirty healthy male Sprague-Dawley (SD) rats at age of 6 to 8 weeks (weighing 180–220 g) were available from the Animal Experimental Center of Southern Medical University, China. The experimental rats were fed for a week under adaptive environmental conditions with 12-h light/dark cycle, standard diet as well as free drinking water. Thirty rats were classified into Control group, Radiation group and Curcumin + Radiation group (10 rats/each group). Rats in the Control group had no irradiation, rats in the Radiation group were treated with 4Gy irradiation, and rats in Curcumin (Cur) + Radiation group were intragastrically administrated with curcumin (99%, Sigma-Aldrich, St. Louis, MO, USA) at dose of 30 mg/kg body weight for 2 weeks once a day after 4Gy irradiation [24, 25]. Radiation procedure: The rats were anesthetized with pentobarbital sodium (50 mg/kg), fixed on the irradiation platform in prone position and irradiated using F992AT type 500 mA X-ray machine (the Radiation Therapy of Jilin Medical College, China). The lead block covered the left lung and mediastinum, and the right lung irradiation field was 2 cm × 3 cm. Rat were irradiated with 6 mV X-ray produced by a linear accelerator [dose: 4Gy, source-to-surface distance (SSD): 100 cm, irradiation dose rate: 600 cGy/min]. The total amount was 12Gy, which was divided into three irradiations (4Gy/each time). The rats woke up naturally after irradiation. Throughout the experiment, the body weight changes of rats were recorded on the 3rd, 7th and 14th days after curcumin gavage.

### Sample collection

After continuously intragastric administration of curcumin for 2 weeks, rats were anesthetized with pentobarbital sodium (50 mg/kg). All animals were euthanized by decapitation, then the vena cava blood was obtained, and centrifuged for 10 min at 3000 g to obtain serum. After blood collection, the liver was quickly obtained, cut into pieces, and preserved at − 80 °C for further use.

### Serum biochemical analysis

The collected serum was thawed at room temperature, and an automatic biochemical analyzer (7080 type, Hitachi, Tokyo, Japan) was utilized to detect total protein (TP), ALT, AST, alkaline phosphatase (ALP), and lactic dehydrogenase (LDH) activity.

### Determination of anti-oxidant factor levels

The liver tissues (200 mg) were homogenized, and centrifuged for 5 min at 1000 g under ice bath to prepare 10% liver tissue homogenate. The homogenate was subjected to centrifugation for 15 min at 1000 g (4 °C) to harvest the supernatant for further biochemical analysis. Malondialdehyde (MDA), catalase (CAT), glutathione (GSH) and superoxide dismutase (SOD) levels were tested in the light of the Kit’s requirements (Jiancheng Bioengineering, Nanjing, China).

### Histological examination

Liver tissues were placed in neutral formalin buffer (10%) for 24 h, dehydrated and embedded in paraffin after routine dehydration, and then sliced into 5 μm sections, followed by dewaxing and hematoxylin-eosin (HE) staining. Subsequently, the pathological morphological changes of liver tissues of rats were viewed using an optical microscope (CX31; Olympus, Tokyo, Japan).

### Transferase-mediated deoxyuridine triphosphate-biotin nick end labeling (TUNEL) staining

The paraffin sections of liver tissues were stained referring to the operation steps of TUNEL kits (Beyotime, Shanghai, China), and cell apoptosis of liver tissues was observed by a light microscope (yellow staining of nucleus was positively stained). Six fields of vision were chosen for each stained section, and total number of the hepatocytes and the positive-stained cells in each field were counted to take the average value. The apoptotic index (%) was calculated as: the positive cells number/ hepatocytes total number) × 100%.

### Western blot analysis

Frozen liver samples were homogenized in chilled radioimmunoprecipitation assay (RIPA) buffer supplemented with protease and phosphatase inhibitors in an ice bath to obtain a liver homogenate. The supernatant was collected by centrifuging the homogenate at 1.4 × 10^3^ r/min for 15 min at 4 °C, and protein concentration was measured using bicinchoninic acid protein detection kits (Beyotime, Shanghai, China). The protein (40 μg) was transferred onto nitrocellulose (NC) membrane by 10% sodium dodecyl sulfate polyacrylamide gel electrophoresis, and blocked with 5% skim milk in tris buffered saline/Tween-20 (TBST). Meanwhile, the membranes were probed at 4 °C overnight with rabbit antibodies against NF-κB p65 (#3034, 1: 1000 dilution), phosphorylated (p)-NF-κB p65 (#3033, 1: 1000 dilution), p-IκB-α (#2859, 1: 1000 dilution), cleaved Caspase-3 (#9661, 1:1000 dilution), Bax (#2772, 1:1000 dilution), glyceraldehyde phosphate dehydrogenase (GPDH; #5174, 1: 1000 dilution), and mouse antibodies against IκB-α (#4814, 1: 1000 dilution) and Bcl-2 (#15071, 1: 1000 dilution). Next, the membranes were detected with secondary antibody (#14708 or #14709, 1: 3000 dilution) after TBST washing. All antibodies were available from Cell Signaling Technology (Danvers, MA, USA). Next, the membranes were tested with an enhanced chemiluminescence (ECL) kit (BIO-RAD, Hercules, CA, USA). The blots were scanned, and the quantification of the band intensity was performed using ImageJ (National Institute of Health, Bethesda, MA, USA).

### Enzyme-linked immunosorbent assay (ELISA)

The contents of interleukin (IL)-1β, IL-6 as well as tumor necrosis factor (TNF)-α in rat liver tissue homogenate were tested with corresponding rat ELISA kits (RAB0277, RAB0311, RAB0479, Sigma-Aldrich, St. Louis., MO, USA), respectively.

### Statistical analysis

Data were processed by Statistical Product and Service Solutions (SPSS) version 21.0 statistical software (IBM Co., Armonk, NY, USA). Before the analysis, the data were checked for normal distribution. The data that conformed to the normal distribution were depicted as mean ± standard deviation (SD), and the analysis of variance (ANOVA) was adopted for comparisons in three or more groups, and the pairwise comparison was implemented with Tukey’s post hoc test. *P* < 0.05 was suggested to be statistically significant.

## Results

### Effect of curcumin on body weight and histopathology in RILD rats

**Fig. 1:**
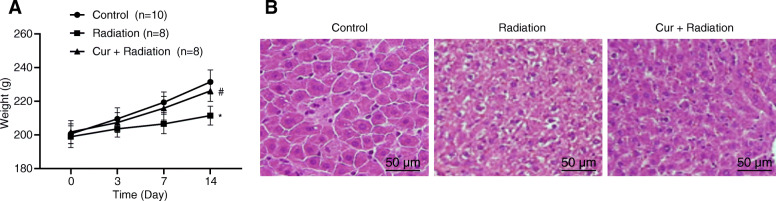
Body weight and pathological changes of rats. **a** Body weight observation in rats. **b** HE staining to view the pathological changes of liver tissues of rats at the 14th day (200 ×). ^*^P < 0.05 vs. Control group vs Radiation group; ^#^*P* < 0.05 Radiation group vs Cur + Radiation group. Cur: curcumin

It was suggested that the rats in the Control group were flexible in their activities and responses, and their water intake was normal. No dead rats were found. The rats in the Radiation group showed decreased activity and food intake, and gradually lost weight. The phenomenon of clustering and hair-raising was more obvious. The fur of rats was tarnished and 2 rats died. In the Cur + Radiation group, there were no obvious symptoms and weight loss, and 2 rats died. As displayed in Fig. 1a, the body weight of rats in the Radiation group on day 14 was lower than that in the Control and Cur + Radiation groups (both *P* < 0.05). Additionally, the results of HE staining of liver tissues under the light microscope were observed as shown in Fig. 1b. In the Control group, hepatocytes of the rats arranged neatly and the hepatic lobule structure was intact, central veins and radial hepatocyte cords were visible. In the Radiation group, hepatic cell edema and inflammation could be visible under the light microscope, and hepatocytes infiltrated and presented with vacuolar degeneration. Compared to the Radiation group, the hepatocytes swelled under the microscope, and the pathological changes were alleviated in the Cur + Radiation group.

### Curcumin ameliorates live function of rats with RILD

**Fig. 2:**
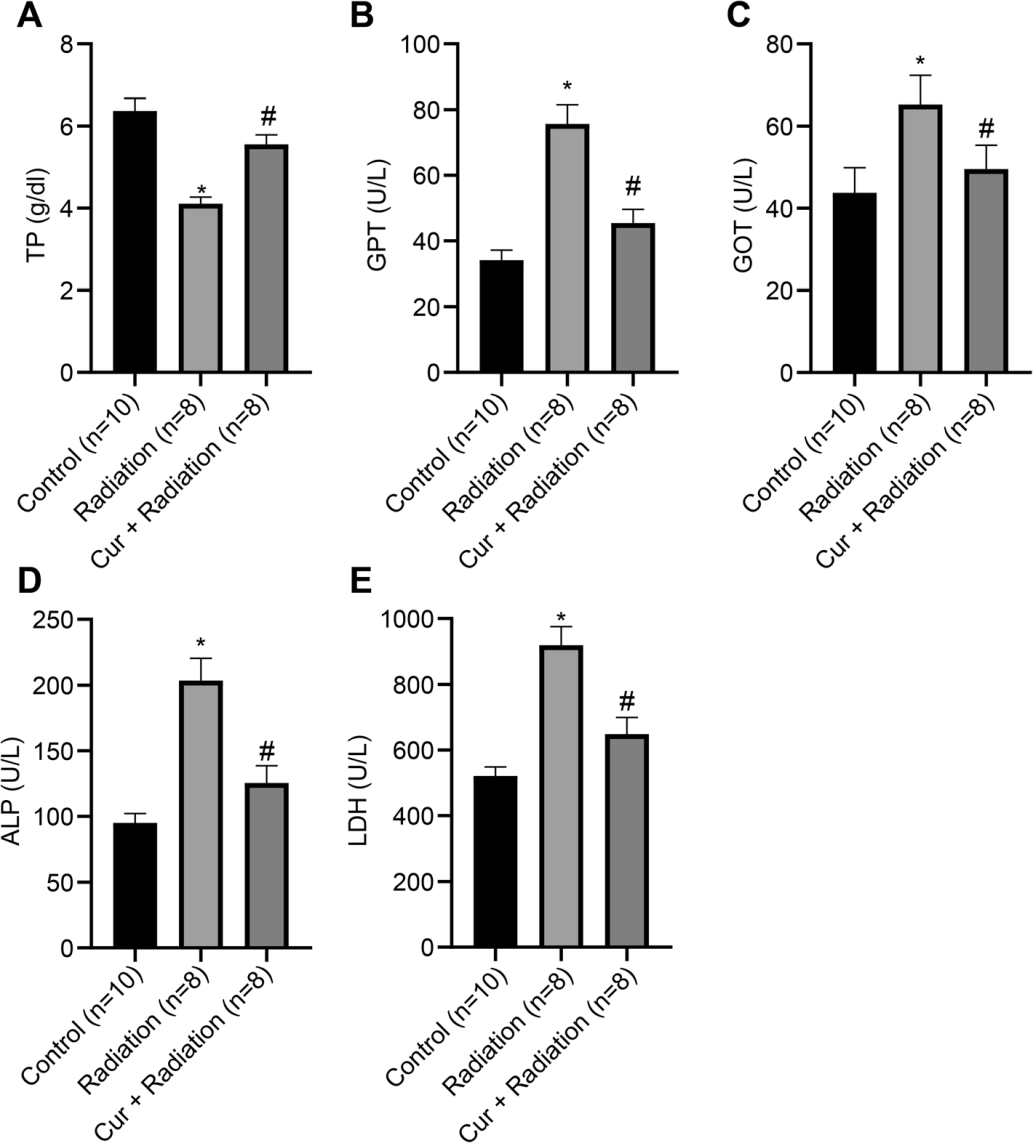
Findings of serum biochemical indices in rats. **a** Detection of serum TP index in rats. **b** Detection of serum ALT index in rats. **c** Detection of serum AST index in rats. **d** Detection of serum ALP index in rats. **e** Detection of serum LDH index in rats. ^*^P < 0.05 Control group vs Radiation group; ^#^*P* < 0.05 Radiation group vs Cur + Radiation group. Cur: curcumin; TP: total protein; ALT: alanine aminotransfetase; AST: aspartate aminotransfetase; ALP: alkaline phosphatase; LDH: lactic dehydrogenase

The serum biochemical indices TP, ALT, AST, ALP, LDH of rats were measured using an automatic biochemical analyzer. It was shown that, compared to the Control group, TP level (Fig. 2a) was reduced, while ALT (Fig. 2b), AST (Fig. 2c), ALP (Fig. 2d) and LDH levels (Fig. 2e) were increased in the Radiation group (all *P* < 0.05). Moreover, rats treated with curcumin exhibited increased TP level and decreased ALT, AST, ALP, and LDH activities in contrast to the Radiation group (all *P* < 0.05).

### Curcumin reduces oxidative stress in rats with RILD

**Fig. 3:**
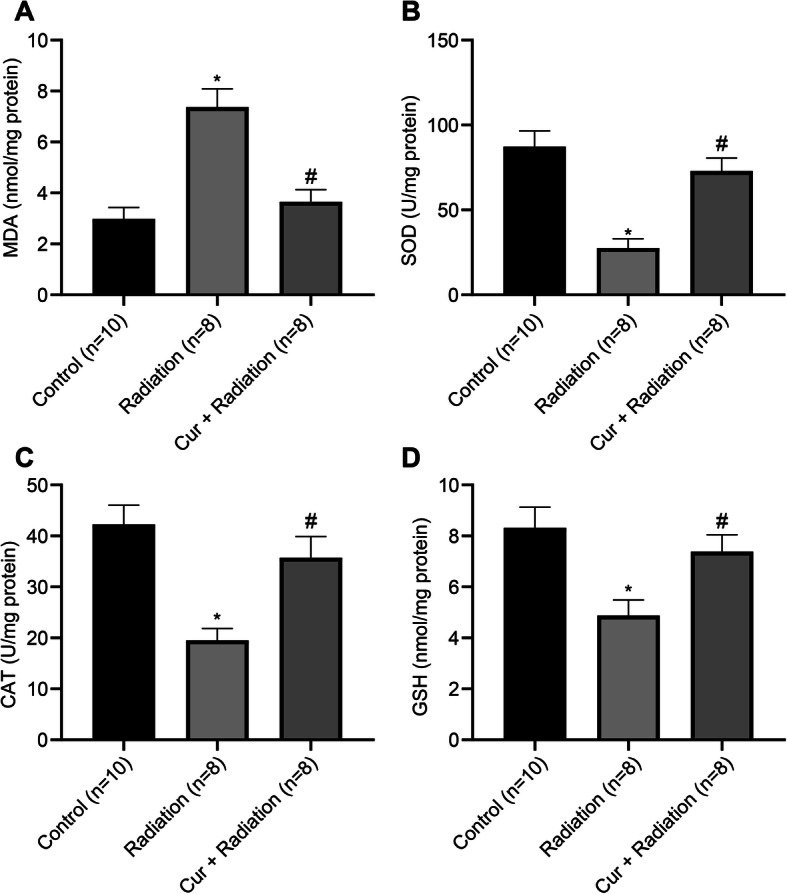
Detection of oxidative stress-related indices in rats. **a** Detection of MDA level in liver tissue homogenate of rats. **b** Detection of SOD level in liver tissue homogenate of rats. **c** Detection of CAT level in liver tissue homogenate of rats. **d** Detection of GSH level in liver tissue homogenate of rats. ^*^P < 0.05 vs. Control group vs Radiation group; ^#^*P* < 0.05 Radiation group vs Cur + Radiation group. Cur: curcumin; MDA: malondialdehyde; SOD: superoxide dismutase; CAT: catalase; GSH: glutathione

Compared to the Control group, the MDA level (Fig. 3a) in the rat liver tissues of the Radiation group was elevated, while the SOD (Fig. 3b), CAT (Fig. 3c), and GSH (Fig. 3d) levels were reduced (all *P* < 0.05). Reduced MDA level, and elevated SOD, CAD and GSH levels were observed in the Cur + Radiation group compared to the Radiation group (all *P* < 0.05; Fig. 3).

### Curcumin reduces the apoptosis in rats with RILD

**Fig. 4:**
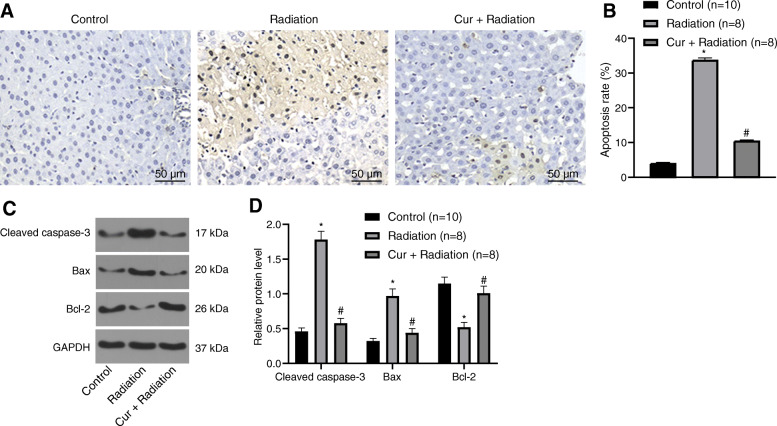
Detection of apoptosis level in the liver tissues of rats. **a** The apoptosis level in the liver tissues of rats was determined by TUNEL staining (200 ×). **b** Statistical results of Panel **a**. **c** Western blot analysis for the determination of apoptosis-related proteins in rat liver tissues. **d** Grayscale statistics of Panel **c**. ^*^P < 0.05 Control group vs Radiation group; ^#^*P* < 0.05 Radiation group vs Cur + Radiation group. Cur: curcumin

To understand whether curcumin oral gavage has anti-apoptotic effects, TUNEL staining together with Western blot analysis were adopted to detect apoptosis-related proteins in liver tissues after 14 days of curcumin oral gavage. The apoptotic rate of liver tissues in the Radiation group was elevated in comparison to the Control group. However, it was obviously diminished in the Cur + Radiation group compared to the Radiation group (all *P* < 0.05; Fig. 4a, b). Further, compared to the Control group, Caspase-3 and Bax expression was increased while Bcl-2 expression was reduced in the liver tissues of rats in the Radiation group. Additionally, Caspase-3 and Bax expression was reduced while Bcl-2 expression was elevated in the Cur + Radiation group versus the Radiation group (all *P* < 0.05; Fig. 4c, d).

### Curcumin reduces inflammation in rats with RILD

**Fig. 5:**
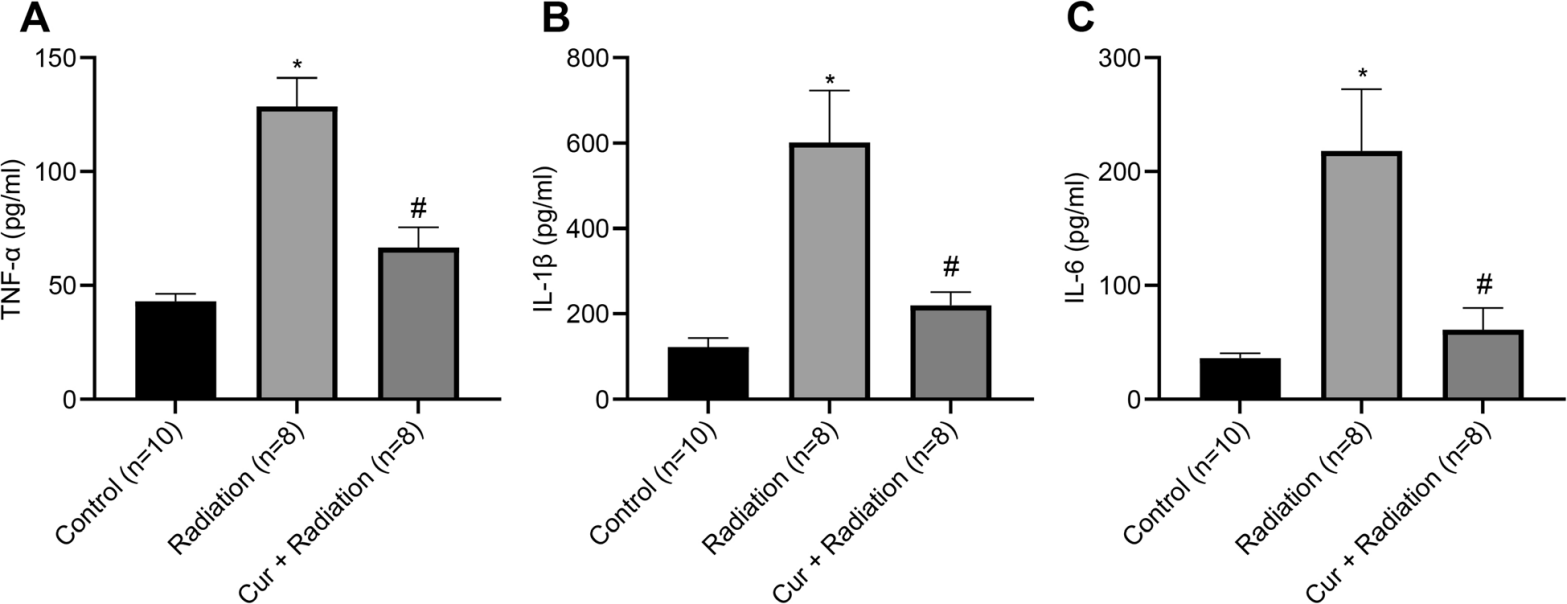
Detection of inflammatory factors in liver tissues of rats. **a** ELISA was utilized to measure TNF-α level in liver tissues of rats. **b** ELISA was utilized to measure IL-1β level in liver tissues of rats. *C. ELISA* was utilized to measure IL-6 level in liver tissues of rats. ^*^P < 0.05 vs. Control group vs Radiation group; ^#^*P* < 0.05 Radiation group vs Cur + Radiation group. Cur: curcumin

The levels of TNF-α, IL-1β, and IL-6 in three groups were tested by ELISA (Fig. 5). Compared to the Control group, TNF-α (Fig. 5a), IL-1β (Fig. 5b), and IL-6 (Fig. 5c) levels were elevated in the liver tissues of rats in the Radiation group (all *P* < 0.05). TNF-α, IL-1β, and IL-6 levels were reduced in the Cur + Radiation group versus the Radiation group (all *P* < 0.05).

### Curcumin inhibits the NF-κB pathway in RILD rats

**Fig. 6:**
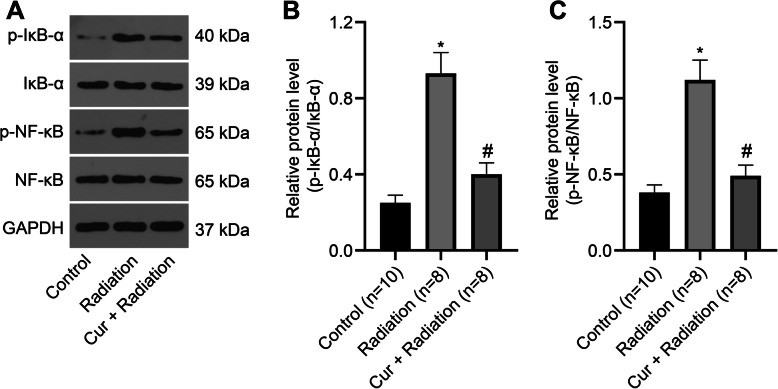
Curcumin inhibits the NF-κB pathway activation. **a** Western blot analysis for the determination of NF-κB pathway protein expression in rat liver tissues. **b** The p-IκB-α/IκB-α level in rat liver tissues. **c** The p-NF-κB/NF-κB level in rat liver tissues. ^*^P < 0.05 vs. Control group vs Radiation group; ^#^*P* < 0.05 Radiation group vs Cur + Radiation group. Cur: curcumin

Also, we measured the changes of proteins on NF-κB pathway by Western blot analysis (Fig. 6a). The findings revealed that p-IκB-α/IκB-α (Fig. 6b) and p-NF-κB/NF-κB (Fig. 6c) protein expression levels were upregulated in the Radiation group versus the Control group. At 14 days after intragastric administration of curcumin, p-IκB-α/IκB-α and p-NF-κB/NF-κB protein expression levels were reduced (Fig. 6).

## Discussion

Radiation-induced liver damage (RILD) usually occurs due to the radiotherapy of hepatocellular carcinoma, and it is a chronic or subacute liver damage in clinic [26, 27]. In addition, RILD severely restricts the reirradiation of hepatobiliary tumors and the elevation of the tumor radiotherapy dose [28, 29]. Curcumin, as a naturally-occurring phenolic compound [30], has been found to be significant in the suppression of tumorigenesis, oxidation, platelet aggregation, or inflammation [31]. Meanwhile, curcumin is demonstrated to have other protective roles against various diseases [32–34]. The obtained results of this article indicate that curcumin treatment reliefs the liver damage caused by radiation through inhibiting the NF-κB signal pathway.

Oxidative stress has been considered a key causing factor of liver damage. The ionizing radiation-induced oxidative stress is caused by increased free radicals’ production which can attack different components in the cells, resulting in biochemical changes and macromolecule modifications [35, 36]. The protective effect of curcumin against oxidative stress was previously described both in vitro and in vivo [37]. For instance, curcumin can be used as a radio protective agent due to its ability to reduce oxidative stress and inhibit transcription of genes related to oxidative stress and inflammatory responses [38]. Most of the toxic effects of ionizing radiation to normal tissue are due to the generation of ROS which triggers formation of several reactive intermediates. To overcome such events, living cells are equipped with integrated endogenous enzymatic and antioxidant systems such as SOD, CAT, GPx and GSH [39]. In line with our findings, it is reported that curcumin pretreatment to γ-induced hepatocytes leads to reduced lipid peroxidation and alleviated anti-oxidant status, thereby hampering its damage to the hepatocytes [40]. Another study revealed that curcumin pretreatment protects against γ-irradiated damage through suppressing membrane lipids peroxidation and DNA strand break formation induced by free radicals [41]. In this study, it is found that curcumin protects liver functions and alleviates oxidative stress of rats from radiation damage through the changes of ALT, AST, ALP, LDH, MDA, TP, SOD, CAD and GSH levels.

Our study also suggests that curcumin contributes to the prevention of inflammation in rats with RILD. Inflammation induced by deleterious circumstances is the product of complex series of responses triggered by the immune system, and it also causes a wide range of physiological and pathological morbidities [42]. Changes of inflammation are indicated by the increase of pro-inflammatory cytokines such as IL-6, IL-1 and TNF-α, genes encoded by activation of NF-κB [43]. Curcumin alleviates inflammation in chronic diseases and regulates inflammatory and pro-inflammatory pathways related with most chronic diseases [44]. Curcumin was reported to be useful in management of inflammation in non-alcoholic fatty liver disease (NAFLD) [45]. Wang et al. [34] have proposed that post curcumin treatment alleviates the severity of arthritis and the histopathological changes, and decreases proinflammatory cytokines release in rats with collagen-induced arthritis [46]. This study finds that curcumin reduces inflammation in rats with RILD through suppression of TNF-α, IL-1β, and IL-6 secretion.

The in vitro and in vivo studies suggested that curcumin alleviates oxidative stress, inflammation in chronic diseases through the Nrf2-keap1 pathway, the production of TNF and the cell signaling mediated by TNF [43]. As previously described, the NF-κB pathway is depicted to be the most important pathway for curcumin-regulated oxidative stress [47, 48]. Curcumin induces cell death of liver cancer stem cells (CSCs) or exerts its therapeutic effects on hepatitis B virus infection by directly targeting the NF-κB pathway [49, 50]. In this study, it is shown that curcumin attenuates radiation-induced liver damage by blocking the NF-κB pathway. A previous study proved that NF-κB activation restricts the apoptotic response to radiation, which offers a mechanism for evading potential cytotoxicity and antiangiogenic effects of tumor microvasculature to radiation therapy [51]. In an article reported by He and his colleagues, curcumin is found to be beneficial in the prevention of inflammation and oxidative damage through activating the NF-κB pathway in the liver [52]. Curcumin has been revealed to down-regulate the NF-κB pathway activation, which is modulated by the decrease of IκB kinase and p-IκBα, thereby inhibiting proliferation and promoting apoptosis of cells [53]. Cao et al. [54] observed that NF-κB expression downregulated by curcumin is in line with its inhibition of the expression levels of proinflammatory cytokines, implying that curcumin modulates cytokine secretion through the NF-κB pathway. In addition, curcumin treatment has been demonstrated to decrease the pro-inflammatory cytokines, oxidative stress, and other chemokines in NASH mice, while NF-κB is attenuated by curcumin treatment in NASH [6]. This study further provides the detailed relationship between curcumin and the NF-κB pathway in RILD rats.

## Conclusion

In summary, it is highlighted that curcumin treatment protects rats with RILD by reducing oxidative stress, inflammation as well as apoptosis via the modulation of the NF-κB pathway. Hence, curcumin administration before radiation therapy can be helpful to prevent hepatocyte damage.

## Supplementary Information

**Additional file 1.**

**Additional file 2.**

## Data Availability

The datasets used or analyzed during the current study are available from the corresponding author on reasonable request.

## Abbreviations

RILD: Radiation-induced liver damage
TP: Total protein
ALP: Alkaline phosphatase
ALT: Alanine aminotransfetase
LDH: Lactate dehydrogenase
AST: Aspartate aminotransfetase
HE: Hematoxylin-eosin
TUNEL: Transferase-mediated deoxyuridine triphosphate-biotin nick end labeling
NF-κB: Nuclear factor kappa B
RIPA: Radioimmunoprecipitation assay
SDS-PEGE: Sodium dodecyl sulfate polyacrylamide gel electrophoresis
TBST: Tris buffered saline/Tween-20
ELISA: Enzyme-linked immunosorbent assay
IL: Interleukin
TNF: Tumor necrosis factor
CCl_4_: Tetrachloride
NASH: Non-alcoholic steatohepatitis
STAT3: Activator of transcription 3
MAPK: p38 mitogen-activated protein kinase
Nrf2: Nuclear factor E2-related factor 2
ROS: Reactive oxygen species
COX-2: Cyclooxygenase-2
VEGF: Vascular endothelial growth factor
SPSS: Statistical Product and Service Solutions
SD: Standard deviation
ANOVA: Analysis of variance
