# Supplementary figures and images for: Curcumin protects radiation-induced liver damage in rats through the NF-κB signaling pathway
Source: BMC Complement Med Ther. 2021 Jan 6;21:10. doi: 10.1186/s12906-020-03182-1 (PMC7789609; doi:10.1186/s12906-020-03182-1)

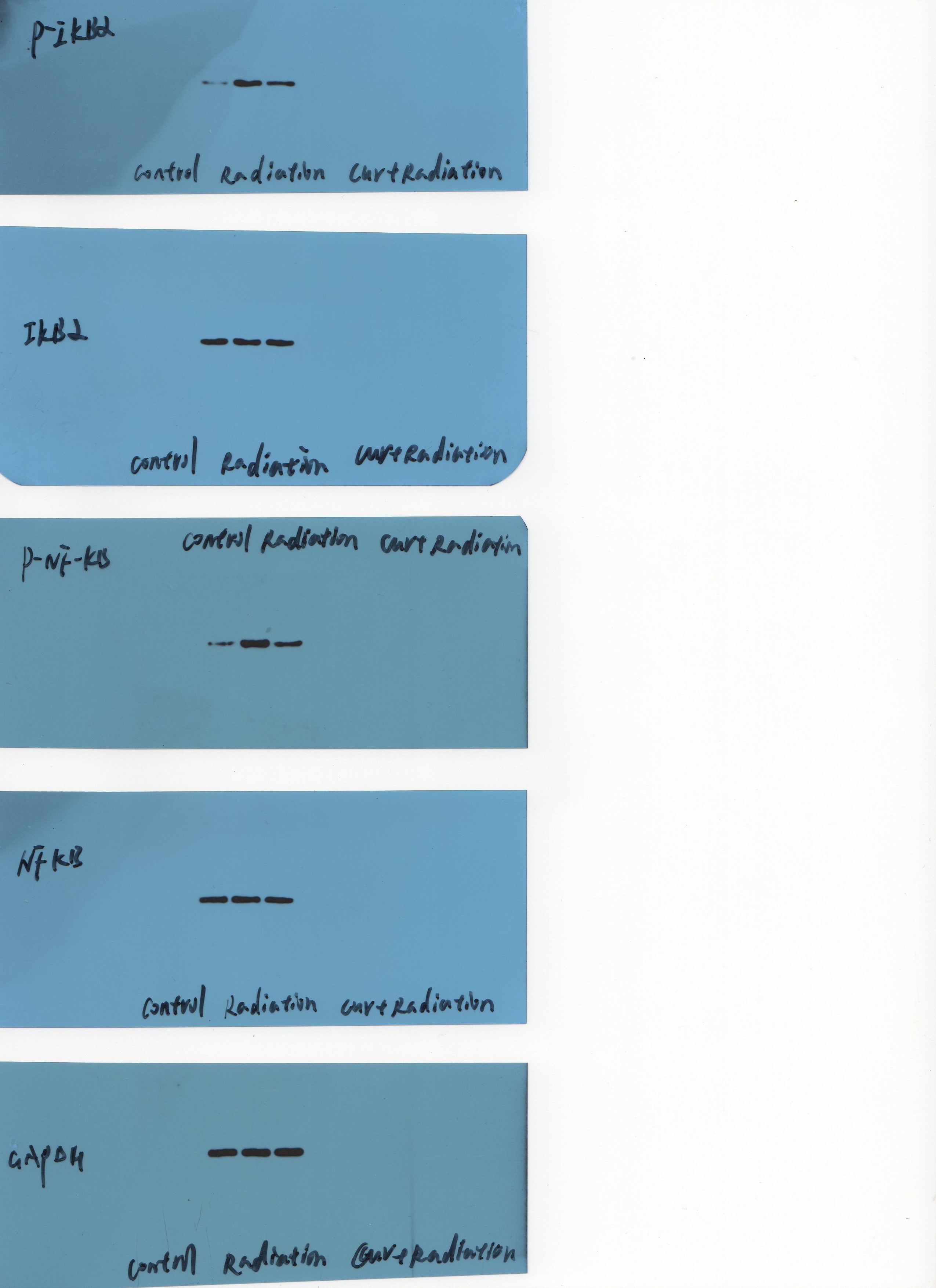

Supplement: Supplementary file 1 — Additional file 1. [file 12906_2020_3182_MOESM1_ESM.jpg]

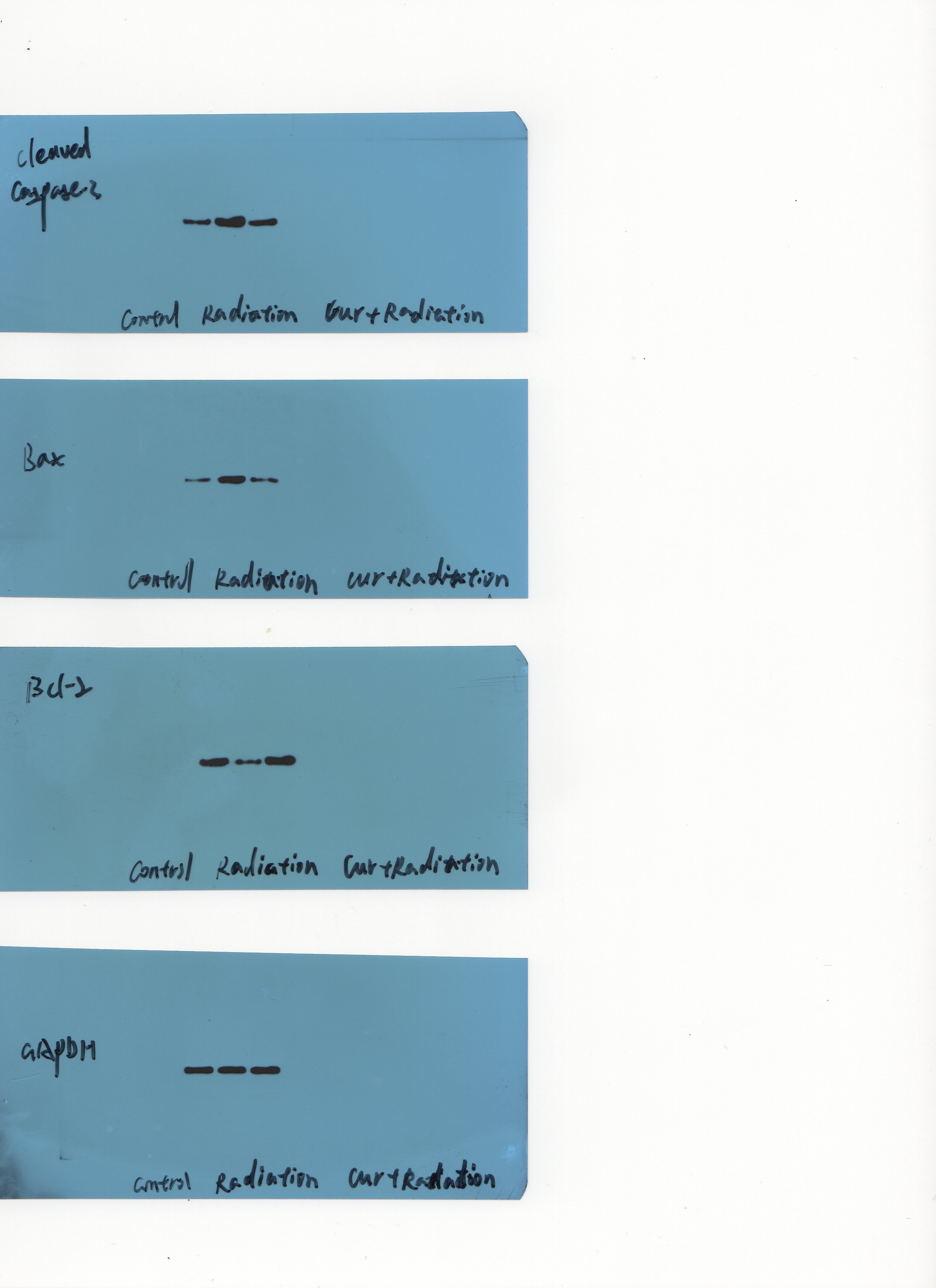

Supplement: Supplementary file 2 — Additional file 2. [file 12906_2020_3182_MOESM2_ESM.jpg]
